# Overexpression of chemokine ligand 7 is associated with the progression of canine transmissible venereal tumor

**DOI:** 10.1186/1746-6148-8-216

**Published:** 2012-11-09

**Authors:** Hsin-Chien Chiang, Yu-Shan Wang, Chung-Hsi Chou, Albert Taiching Liao, Rea-Min Chu, Chen-Si Lin

**Affiliations:** 1 Animal Cancer Center, School of Veterinary Medicine, National Taiwan University, Taipei, Taiwan; 2 Department of Radiation Therapy and Oncology, Shin Kong Wu Ho-Su Memorial Hospital, Taipei, Taiwan; 3 School of Veterinary Medicine, National Taiwan University, Taipei, Taiwan

**Keywords:** CXCL7, CXCR2, IL-6, TGF-β, CTVT

## Abstract

**Background:**

Chemokines play multiple roles in the development and progression in a variety of tumors. Chemokine (C-X-C motif) ligand 7 (CXCL7) has been found associated with pro-inflammatory responses, but its role in cancer growth remains unclear. Our previous study showed that R phase tumor infiltrating lymphocytes (TILs) produced large amounts of interleukin (IL)-6 which antagonized transforming growth factor (TGF)-β derived from CTVT to diminish the immune-suppressive microenvironment. Now we intend to determine the expression pattern of CXCL7 and the role of IL-6/TGF-β in CXCL7 induction during spontaneous progressive (P) and regressive (R) phases in canine transmissible venereal tumor (CTVT).

**Results:**

We have demonstrated that CXCL7 expressed at high level in P phase and down-regulated in R phase by western blot and real-time PCR. This suggested that CXCL7 expression was negatively correlated with the tumor growth. Co-culturing TILs with CTVT cells was found to reduce CXCL7 expression, while adding IL-6 blocking antibody reversed it. Moreover, in P phase CTVT, while IL-1β and TGF-β had no obvious effect on CXCL7 expression, IL-6 was found significantly to reduce CXCL7 expression in a dose-dependent manner. The mRNA expression results of CXCL7 receptor, CXCR2, further confirmed the effects of IL-6 concentration on the CXCL7 expression.

**Conclusion:**

CXCL7 overexpression might be associated with the progressive growth of CTVT. The results shown here also suggest the role of CXCL7 in cancer development and the potential as the anti-cancer therapeutic target.

## Background

Canine transmissible venereal tumor (CTVT) is a unique tumor that is transmitted by viable tumor cells [1] through injured mucosa and skin. CTVT cells have a stabilized genome with almost identical dog leukocyte antigen class II loci, which may have originated in wolves [1]. The tumor cells effectively evade the host’s histocompatibility (MHC) barrier for long periods, and transplanted cells grow liberally in the progression (P) phase for a few months or over 1 year [2,3]. This immunoevasion was found partly because of the high concentration of tumor-secreted Transforming growth factor-β (TGF-β), which inhibits tumoral MHC antigen expression and the activity of natural killer cells [4]. However, the immune systems of most hosts eventually reject the transplanted cells in the regression (R) phase [5]. One mechanism for this rejection is related to interleukin (IL)-6 produced by tumor-infiltrating lymphocytes (TIL) that counteract the activities of TGF-β [4]. CTVT is one of the few tumors that allow us to study detailed dynamic changes in host–cancer interactions during spontaneous regression of a tumor.

Chemokines are classified as a group of 8–10 kDa proteins with structural similarity and chemotactic activity [6]. Chemokines are generated by leukocytes and several types of stromal cells and are chemoattractants for a wide range of leukocytes. Chemokines play an important role in lymphocyte migration, recirculation, modulation of immune response, and leukocyte recruitment to the inflammation sites. There are in the main two subfamilies of chemokines, which are defined based on the arrangement of the first two cysteines, which can be either adjacent (C-C) or separated by one other amino acid (C-X-C). CXC chemokines act on neutrophils, and maintain the amino-terminal sequence Glu-Leu-Arg (ELR) motif, which is necessary for receptor recognition and signaling [7]. One of the ELR^+^CXC chemokines, Chemokine (C-X-C motif) ligand 7 (CXCL7), has been shown to activate neutrophils for chemotaxis over a wide range of stimulus concentrations [8], for the degranulation of lysosomal enzymes [9] and to increase adhesion molecules on these cells [10]. Activated CXCL7 induces the recruitment of neutrophils to the site of inflammation or injury. Increased CXCL7 levels were detected in epithelial cells isolated from clinical patients with active ulcerative colitis, which suggests that CXCL7 contributes to the sustained neutrophil presence in these patients. However, unlike other ELR^+^CXC chemokines [11-13], the relationship between CXCL7 and cancer has not been well investigated.

In the tumor microenvironment, chemokines and their receptors play pivotal roles in regulating angiogenesis, cell migration and tumor growth and help to clarify the progression of the cancer [14]. Some particular chemokine–receptor pairs are included in tumor metastasis by their chemoattractants function in cell migration. In addition, chemokines make the tumor microenvironment more suitable for tumor growth by induction of leucocytes migration and activation of inflammatory responses [14]. Recently accumulated evidence shows that chemokine receptor signaling also contributes to tumor survival and proliferation, mainly due to their migratory promotion activities [15]. Many studies have shown that CXC chemokines are co-expressed in a variety of tumors [11-13] and account for the higher aggressiveness of tumors [11].

Our previous study showed that canine dendritic cells (DC) derived from peripheral blood express CXCL7 mRNA and secrete CXCL7 protein in response to chronic inflammation inducing cytokines, such as IL-1β, IL-6, TGF-β and Tumor necrosis factor (TNF)-α, but not to acute inflammation-associated cytokines, such as Interferon (IFN)-γ and lipopolysaccharides (LPS) [16]. These findings suggest that DC produce CXCL7 in a chronic inflammation site under an immune tolerance situation. We also demonstrated that only an immunologically-favorable microenvironment can fully support the maturation of DC [17], and CTVT have the ability to induce an immune escape microenvironment [4,18,19]. Therefore, we hypothesized that the tumor microenvironment of CTVT may involve CXCL7 expression and allow CXCL7 to achieve chronic inflammation and tumor progression.

The relationship between CXCL7 and CTVT is investigated in this study. It was found that CXCL7 overexpression was associated with the progression phase of CTVT, while during the regression phase, the tumor cells express a very low level of CXCL7. In addition, we also revealed that TIL-derived IL-6 down-regulated CXCL7 expression in CTVT. These results suggest a novel role of CXCL7 in cancer progression.

## Results

### Association between CXCL7 overexpression and tumor growth pattern

**Figure 1:**
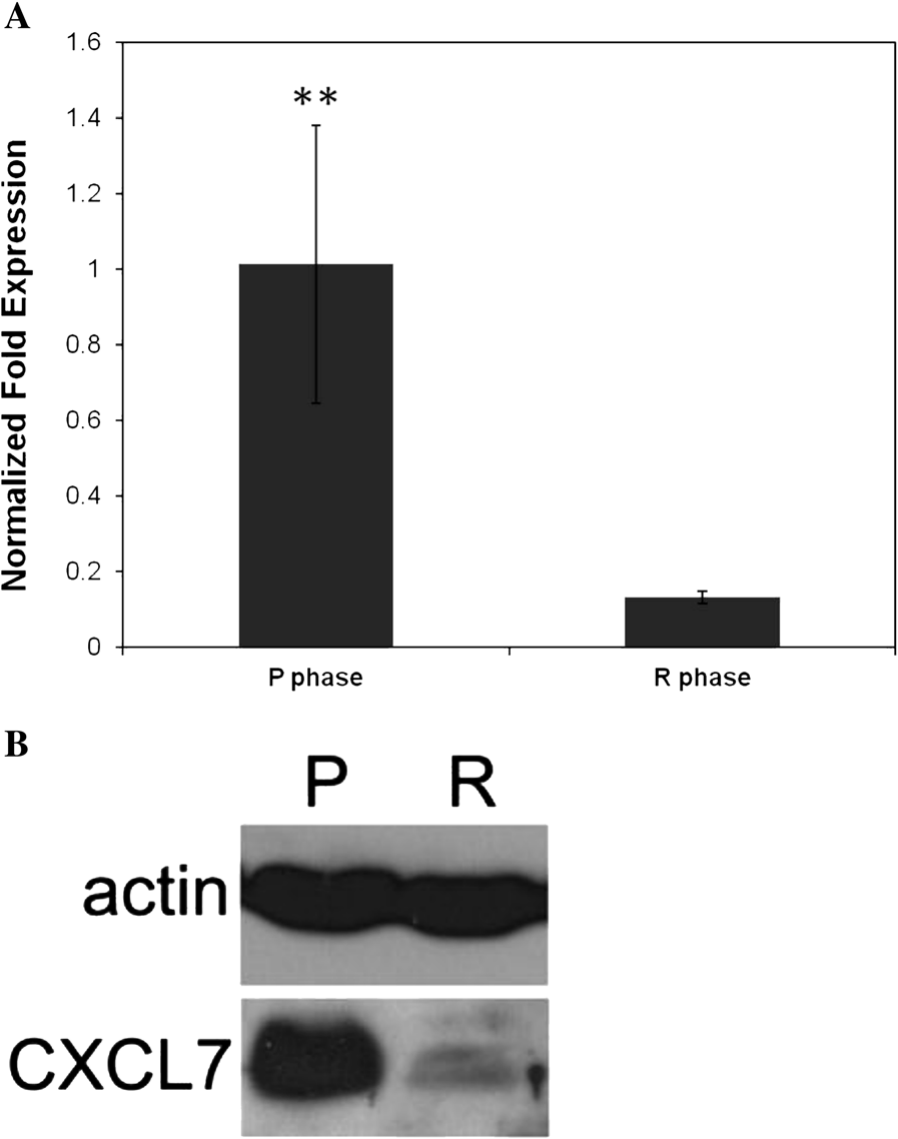
**CXCL7 expression is concordant with the CTVT progression phase.A**. Levels of mRNA expression of CXCL7 from CTVT tumor masses (n = 4) measured by real-time RT-PCR. The amounts of mRNA are expressed relative to the amount of β-actin mRNA in each sample and are shown as the mean ± S.D. (**, *P* < 0.01). **B**. Western blot analysis of CXCL7 protein expressed in CTVT. CXCL7 proteins were recognized by polyclonal rabbit antibody against canine CXCL7 in the lysates of CTVT tumor masses. Actin was used as the internal control.

The mRNA levels of CXCL7 in the tumor masses from the P and R phase were determined by real-time RT-PCR. As shown in Figure 1A, the CXCL7 mRNA expression was significantly higher in the P phase than in the R phase (*P* < 0.01). Western blotting revealed that in comparison with the R phase, the CXCL7 protein was significantly up-regulated in the CTVT P phase (Figure 1B). The specific bands were normalized with the internal control β-actin. These data show that CXCL7 increased concordantly with CTVT progressive growth both in terms of the mRNA and protein expression levels.

### R-phase TIL inhibited the expression of CXCL7 in P phase CTVT cells

**Figure 2:**
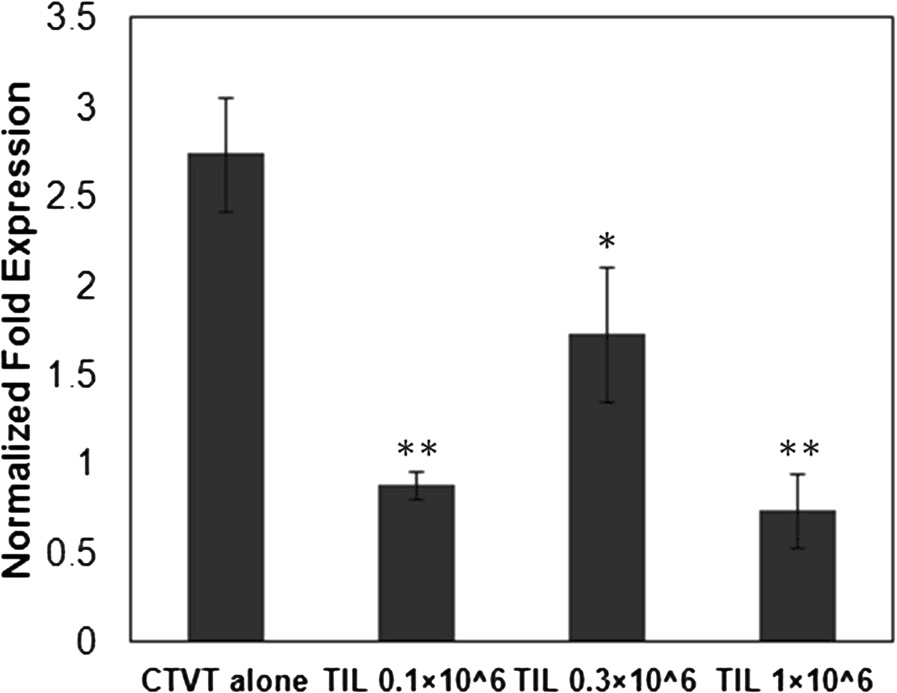
**R-phase TIL inhibited the expression of CXCL7 in P-phase CTVT cells.** A coculture system was analyzed using a Corning transwell system, in which TIL cells (0.1–1×10^6^ cells/well) were seeded in the upper chamber, with a 0.4-μm-pore filter between that and a lower chamber containing CTVT cells (1×10^6^ cells/well). After 12 h of incubation, the gene expression of CXCL7 in the CTVT cells was evaluated. Significant differences in the various stimulators are expressed as * (*P* < 0.05) and ** (*P* < 0.01) as compared with CTVT alone. The results represent the mean ± S.D. of three independent experiments.

To find the factor that regulated the expression of CXCL7, we cocultured P-phase CTVT cells with R-phase TIL using a transwell system. The P-phase CTVT cells were separated from R-phase TIL using the 0.4 μm pore size of the transwell system. After 12 h coculture, the CXCL7 expression was significantly decreased (Figure 2). The inhibitory effect could be achieved even in 1:10 ratio of TIL: CTVT cells. This result suggested that TIL might secrete some soluble substances to inhibit the production of CXCL7.

### The expression of CXCL7 was regulated by IL-6 in CTVT cells

**Figure 3:**
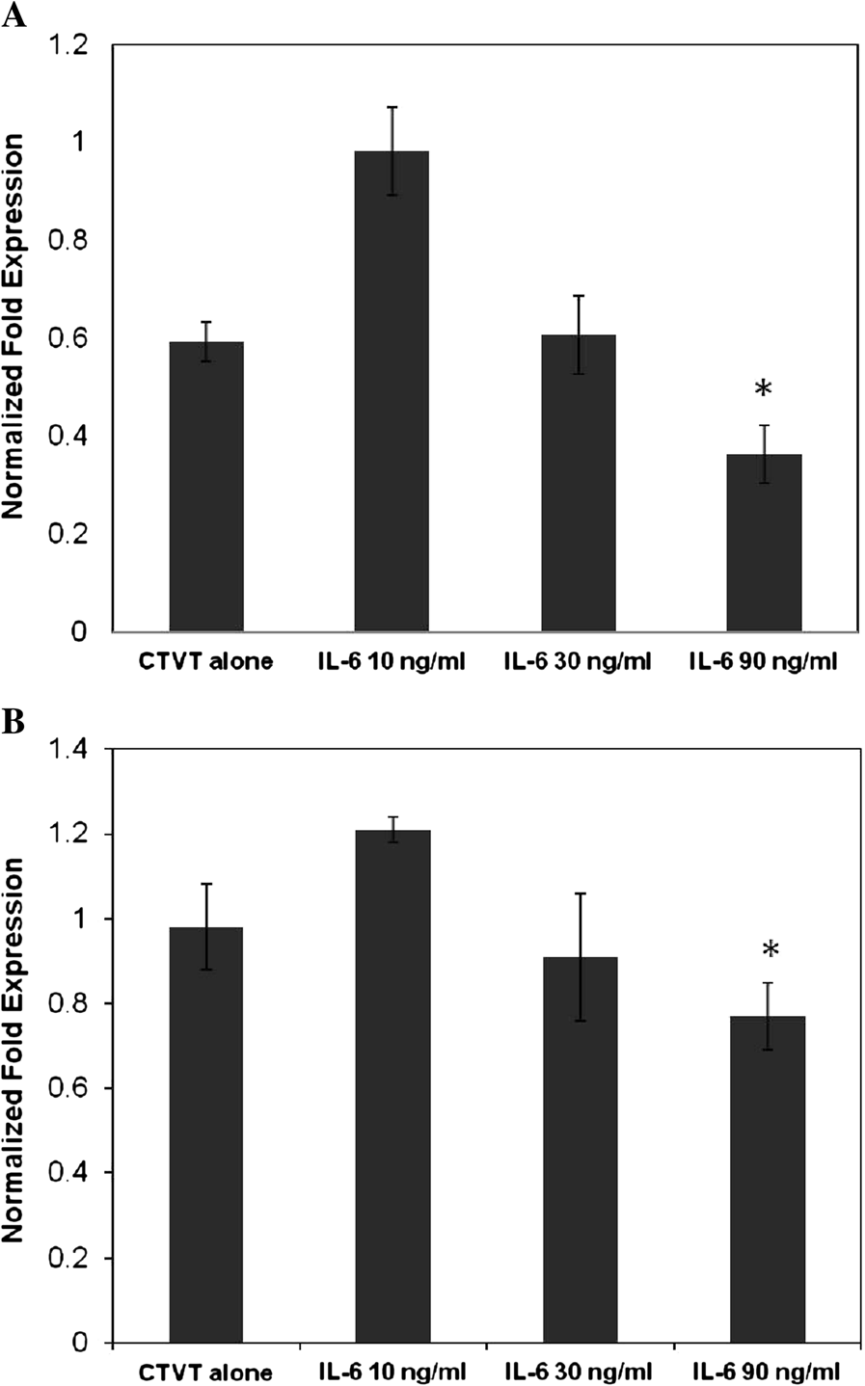
**CXCL7 and CXCR2 expression in P-phase CTVT cells was regulated by IL-6.** Levels of mRNA expression of CXCL7 (**A**) and CXCR2 (**B**) after freshly prepared P phase CTVT cells were treated with 10, 30 or 90 ng/mL IL-6 for 12 h, measured by real-time RT-PCR. The results represent the mean ± S.D. of three independent experiments (* *P* < 0.05 as compared with the CTVT alone group).

As the IL-6 produced by R-phase TIL counteracts the activities of TGF-β to induce CTVT regression [5], we further confirmed that the expression of CXCL7 was regulated by IL-6. The responses of the expression of CXCL7 and its receptor, CXCR2, to different doses of IL-6 were studied. The expression pattern of CXCL7 (Figure 3A) coincided with that of CXCR2 (Figure 3B). CXCL7 and CXCR2 were down-regulated with high-dose IL-6 treatment in a dose response fashion. However, when the concentration of IL-6 was low, such as 10 ng/mL, the CXCL7 production was higher than that in tumor cells without any IL-6 treatment (Figure 3).

### Regulation of CXCL7 expression in CTVT cells

**Figure 4:**
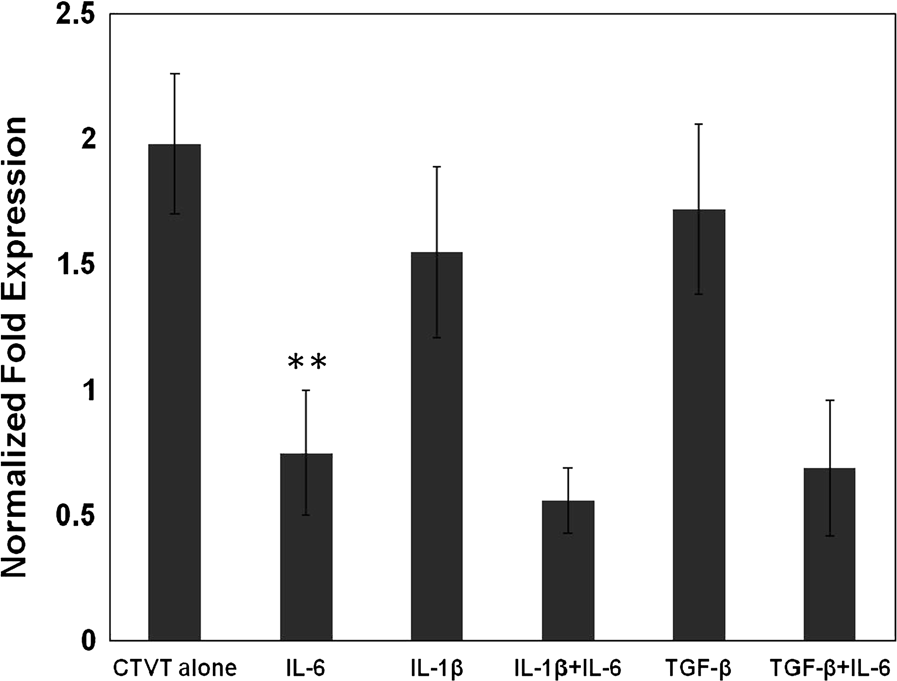
**CXCL7 expression in CTVT under IL-1β, TGF-β and IL-6 stimulation.** Levels of mRNA expression of CXCL7 after freshly prepared P-phase CTVT cells were treated with IL-1β, TGF-β and/or IL-6 (90 ng/mL) for 12 h, measured by real-time RT-PCR. The amounts of mRNA are expressed relative to the amount of β-actin mRNA in each sample and are shown as the mean ± S.D. (**B**) 1 μg/ml of anti-IL-6 antibody was adding into the TIL/CTVT cocultured transwell system. TIL cells (0.3 × 10^6^ cells/well) were seeded in the upper chamber and the lower chamber containing CTVT cells (1 × 10^6^ cells/well). After 12 h incubation, the CXCL7 gene expression in the CTVT cells was evaluated. Significant differences in the various stimulators are expressed as ** (*P* < 0.01) as compared with CTVT alone.

To further investigate the expression of CXCL7 in CTVT, we aimed to examine endogenous signals that up- or down-regulate CXCL7 expression. IL-1β is a common cytokine that stimulates CXCL7 expression [20]. In our previous study, we found that TGF-β was elevated in the CTVT P phase and decreased in the R phase, and TGF-β activities were regulated by IL-6 released from TIL [5]. Based on these observations, we used IL-1β and TGF-β to verify the up-regulation of CXCL7 and used IL-6 to investigate the inhibition of CXCL7 expression in CTVT cells. As shown in Figure 4, IL-1β and TGF-β did not increase the CXCL7 expression in comparison with CTVT cells alone while IL-6 significantly down-regulated the expression of CXCL7 in tumor cells. In addition, the inhibition effect of IL-6 was not influenced by the combination of IL-1β and TGF-β. The IL-6-mediated CXCL7 decrease could be further demonstrated since blocking IL-6 activity in TILs significantly reversed this reduction (Figure 4B). Together, these results indicated that the down-regulation of CXCL7 in the R phase may due to the IL-6 produced by TIL.

## Discussion

The importance of CXCL7 in regulating tumorogenesis in colon cancer has been reported [20]. Our current observations provide further insight into the potential importance of CXCL7 signaling in promoting tumor growth and progression. Several studies have suggested that high expression of ELR^+^CXC chemokines *in vivo* and *in vitro* is associated with a poor prognosis [21,22]. These studies pointed out that high levels of ELR^+^CXC chemokines, including CXCL7, are associated with enhanced tumor growth [20]. High levels of CXC chemokines are associated with risk of recurrence after tumor resection [12]. One report indicated that increasing numbers of tumor infiltrating neutrophils in advanced cancer are associated with reduced mortality [23]. Neutrophils can either exclude tumor cell populations or provide for their invasive potential [24,25]. Neutrophil recruitment by ELR^+^CXC chemokines help tumor cells to metastasize to lymph nodes [12]. Furthermore, ELR^+^CXC also have the ability to stimulate tumor cell proliferation [13]. CXCL7 overexpression in P-phase CTVT confirmed this observation.

In this study, we found that TIL-derived IL-6 played a key role in the modulation of CXCL7 expression in CTVT. The presence of soluble IL-6R in combination with IL-6 affects the recruitment of leukocyte subpopulations through the concurrent ability of the active complex to suppress ELR^+^CXC chemokine expression induced by proinflammatory cytokines (IL-1β and TNF-α) [26]. IL-6 is a pleiotropic cytokine that is produced by many kinds of cells. Depending on the target, IL-6 can suppress growth, elicit growth, or influence differentiation [27,28]. IL-6 can directly elevate the functions of NK cells including proliferation, cytotoxicity, and the expression of surface activation antigen and adhesion molecules [29]. IL-6 and its soluble receptor (sIL-6R) are important in the regulation of leukocyte recruitment [30]. Our data were consistent with these observations. IL-6 could suppress the expression of CXCL7 in CTVT cells. Furthermore, although CTVT-derived TGF-β plays a role in damaging the host immune system during the P phase, TIL-derived IL-6 is responsible for the recovery of immune cells activity during the R phase [18]. Although IL-6 is present in the TIL supernatants of both the P and R phases, its concentration is significantly higher in the R-phase TIL supernatant [5]. This may indicate IL-6 inhibits CXCL7 expression is one of the mechanism responsible for activating host immune system to against tumor. However, the low concentration of IL-6 produced in the P phase may act as an inducer for CXCL7 expression.

Many reasons for the induction of chemokine/receptor expression have been identified. Chemokine receptor expression is regulated both at the transcriptional level and post-transcriptionally via RNA stability, translation and receptor desensitization and internalization [15,31,32]. The tumor microenvironment, and mutant proteins or changed signaling in the tumor cell itself, can also influence the chemokine receptor levels. Conditions present within cancer, such as hypoxia or a rich cytokine environment, can induce the transcription of chemokine receptors [15,32]. The increased expression of CXCR2 in CTVT cells may potentially activate CXCL7 signaling in an autocrine/paracrine pattern, the result of which appears to enhance CTVT cell survival and growth in the host.

In the P phase, IL-1β and TGF-β were expressed at a high level [5]. Tumor-derived TGF-β causes serious immune-inhibition in dogs with CTVT [4,18,19,33]. This encourages us to further investigate the relationship between TGF-β and CXCL7. However, neither IL-1β nor TGF-β could induce greater CXCL7 expression in P-phase CTVT cells (Figure 4). This may be the reason for which freshly isolated CTVT cells alone could express very high levels of CXCL7, so CTVT cells treated with IL-1β or TGF-β failed to express CXCL7 at a higher level than CTVT cells alone. Another possibility is that TGF-β could not induce CXCL7 expression in CTVT cells.

## Conclusion

In conclusion, overexpression of CXCL7 was found to be associated with advanced tumor stage. IL-6 played a modulation role in the expression of CXCL7 in CTVT. Through multiple mechanisms, CXCL7 may be involved in the migration and invasion of cancer cells and in tumor progression. These host–cancer interactions and the mechanisms behind them deserve further study. Another valuable endeavor is research into the mechanisms by which hosts develop efficient ways to defend themselves against tumors that grow over months. Further studies are needed to confirm and understand this interesting observation and to determine whether CXCL7 may serve as a therapeutic target for cancer treatment.

## Methods

### *In vivo* tumor growth

The institutional animal care and use committee of National Taiwan University approved this study before its start. Spontaneous CTVT on the external genitals of a male dog was used for the original transplantation. Six beagles aged 1–1.5 years were used for the experiments. Each beagle was subcutaneously injected with a freshly prepared suspension containing 7.5 × 10^7^ viable tumor cells at eight sites on their backs. The dimensions of the tumors were measured with a caliper once a week, and the volume was estimated as (π × length × width × thickness)/4 in cubic centimeters [5]. A tumor increasing in volume was classified as a P-phase tumor, and one decreasing in volume was classified as an R-phase tumor. MHC expression and the TIL subpopulations of tumors in the P and R phases were analyzed to confirm the growth phases.

### Purification of CTVT cells and tumor infiltrating lymphocytes

Every 2–3 wk post-inoculation, tumor tissue samples were surgically excised from the experimental dogs. The methods previously described were followed to isolate CTVT tumor cells and TIL [4]. Briefly, 10 g of aseptic tumor tissue was minced in 90 mL HBSS (Invitrogen). To obtain a single cell suspension, samples were mechanically crushed with stainless steel mesh and filtered once through two pieces of gauze (pore size: 190 μm). Then, 8 mL of the cell suspension was overlaid on 4 mL 42% Percoll (GE healthcare) gradient and centrifuged at 820 ×*g* and 4°C for 25 min. After centrifugation, CTVT cells deposited at the interface were harvested and washed three times with DMEM (Invitrogen) supplemented with 10% FCS. TIL deposited at the bottom of tube were collected carefully and washed three times with RPMI 1640 (Invitrogen) supplemented with 10% FCS. The purified CTVT cells and TIL were stained with Hemacolor (Merck) to confirm their purities. Freshly isolated CTVT cells and TIL (1 × 10^6^ cells/mL) were cultured at 37°C for future use. To prepare activated CTVT cells, cells were incubated with different concentrations (10, 30 and 90 ng/mL) of IL-6 (Peprotech), 250 pg/mL of TGF-β (R&D systems), and 10 ng/mL of IL-1β (Peprotech) for 12 h, respectively.

### Extraction of RNA from CTVT

Total RNA was extracted from CTVT tumor masses or cells separately with TRIzol (Invitrogen) according to the manufacturer’s recommendations for cultured cells. The amount of total RNA was determined spectrophotometrically at 260 nm.

### Reverse transcription of mRNA into cDNA

Using a SuperScript II reverse transcriptase kit (Invitrogen), 2 μg mRNA were transcribed into cDNA with a final concentration of 5.5 mM MgCl_2_, 0.5 mM of each dNTP, 2.5 μM random hexamers, 0.4 U/μL RNase inhibitor, and 1.25 U/μL multiscribe reverse transcriptase, in a reaction volume of 10 μL. The samples were incubated at 80°C for 10 min, followed by transcription at 37°C for 60 min, and enzyme inactivation at 95° for 5 min.

### Primers for real-time RT-PCR

The primer sequences were designed to bind specifically to canine CXCL7 and CXCR2 cDNA using the Primer3 software, available online (http://frodo.wi.mit.edu/cgi-bin/primer3/primer3_www.cgi), and purchased from Mission Biotech (Taipei, Taiwan). The sequences of the primer pairs were as follows: 5^′^- AGACCTAAGGCCACCTCCTC -3^′^ with 5^′^- GGAACTTCGCTGCATGTGTA -3^′^ for canine CXCL7 cDNA and 5^′^- TCATCTTTGCTGTCGTGCTC -3^′^ with 5^′^- TGTGGAAGAAGCCCAGAATC -3^′^ for canine CXCR2 cDNA. A housekeeping gene, β-actin, was utilized. The GenBank accession numbers of the targets as well as the primer sequences are given in our previous publication [33].

### Real-time RT-PCR

Real-time RT-PCR was performed on an IQ5 real-time PCR detection system (Bio-Rad) using IQ SYBR Green PCR Supermix (Bio-Rad) according to the manufacturer’s guidelines. PCR was conducted in 96-well optical reaction plates as described previously [34]. The relative mRNA amount in each sample was calculated based on the Ct in comparison with the Ct of the housekeeping genes, and the mRNA was ascribed a fold induction of 1. The results are presented as 2^-(Ct of target gene – Ct of housekeeping gene)^ in arbitrary units, as described in a previous publication [33].

### Western blot analysis

CTVT cells were lysed in 250 μL of LaemmLi sample buffer (Bio-Rad) and eluted by 5 min of boiling. Electrophoretic separation of the cell lysates was performed in 15% acrylamide gels, and bands were transferred onto polyvinylidene difluoride (PVDF) membranes (Immobilon-P membrane, Millipore). For immuno-blotting, the membranes were probed with anti-actin mAb (MAB1501, Chemicon International Inc.) at a 1/2500 dilution or rabbit anti-CXCL7 polyclonal antibody (pAb) at a 1/500 dilution. The blots were incubated with rabbit anti-mouse or goat anti-rabbit conjugated HRP secondary antibodies (Jackson ImmunoResearch Laboratories). The signals were revealed by ECL (GE healthcare).

### Statistical analysis

All results are expressed as the mean ± S.D. and were analyzed with a two-tailed Student’s *t* test. Differences were considered statistically significant at *P* < 0.05.

## Competing interest

There is no conflict of interest with any financial organization regarding the material discussed in the manuscript.

## Authors’ contributions

Y-SW established canine DC culture system, found CXCL7 over-expression in P phase CTVT and drafted the manuscript. H-CC followed this discovery and completed the experiments to reveal that TIL-derived IL-6 inhibited CXCL7 expression. C-HC and ATL provided constructive suggestions to fix some minor problems in the study. C-SL and R-MC supervised the whole work and proofread the manuscript. All authors read and approved the final manuscript.
